# Identification of pathological CD133+ endothelial cells in venous malformations

**DOI:** 10.3389/fcvm.2026.1760326

**Published:** 2026-03-30

**Authors:** Carrie J. Shawber, Averill Clapp, Noa Shapiro-Franklin, Shirley Yang, Mason G. Harvill, Emma Iaconetti, Michael J. Schonning, Andrew I. Zeiler, Meghan Perez, Seung Koh, Anna Alkelai, June K. Wu

**Affiliations:** 1 Division of Reproductive Sciences, Department of Ob/Gyn, Columbia University Irving Medical Center Vagelos College of Physicians & Surgeons, and New York Presbyterian/Morgan Stanley Children’s Hospital, New York, NY, United States; 2 Division of Plastic Surgery, Department of Surgery, Columbia University Irving Medical Center Vagelos College of Physicians & Surgeons, and New York Presbyterian/Morgan Stanley Children’s Hospital, New York, NY, United States; 3 Department of Pathology and Cell Biology, Columbia University Irving Medical Center Vagelos College of Physicians & Surgeons, and New York Presbyterian/Morgan Stanley Children’s Hospital, New York, NY, United States

**Keywords:** endothelial cells, progenitor, venous malformations, whole exome sequencing, xenograft

## Abstract

**Introduction:**

Venous malformations (VMs) are congenital malformations of the venous system. Histologically, they are composed of dilated vascular channels. Prior studies have demonstrated that CD31 + endothelial cells (ECs) in VMs have pathogenic variants. Recent studies by our group found that the EC progenitor marker, CD133+, was expressed on VM endothelium in patient tissues. We hypothesized that a CD133+ VM endothelial cells contributes to VM pathobiology.

**Methods:**

VM cells were isolated from resected venous malformation tissues or fluid using CD133 as a marker. Isolated VM populations were characterized by quantative RT-PCR, fluorescence-activated cell sorting (FACS) and immunofluorescence staining (IF) for the expression of progenitor and mature EC genes/proteins. Cells underwent whole exome sequencing (WES) to probe for genetic variants. AKT and ERK activation status was assessed by Western blot and IF, and cell proliferation determined. Isolated CD133+ cells were xenografted in mice and their ability to recapitulate VM phenotype was assessed by histological analysis, IF and colormetric staining.

**Results:**

CD133+ cells isolated from VMs expressed progenitor and mature EC genes and proteins, and we termed them CD133+ VM endothelial cells (CD133+ VMECs). WES revealed CD133+ VMECs had pathogenic variants and variants of uncertain significance in genes reported in VMs, *PIK3CA* and *TEK*. CD133+ VMECs had increase proliferation and a subset had increase nuclear phospho-AKT. When implanted into a xenograft model, CD133+ VMECs with *PIK3CA* and *TEK* variants recapitulated clinical VM phenotypes.

**Conclusion:**

We have identified a novel cell type in VMs, CD133+ VMECs that express EC progenitor proteins, demonstrating incomplete or misdirected differentiation down the EC lineage and are capable of recapitulate the phenotype in a mouse model.

## Introduction

Venous malformations (VMs) are slow-flow malformations of the venous vasculature and originate during vascular development. VMs consist of numerous dilated, ectatic veins with abnormal perivascular cell coverage (1–3). VMs are chronic conditions that can be associated with life-threatening and costly morbidities, including hemorrhage, coagulopathy, and organ obstruction (4–7). Pathogenic variants in genes that activate the PI3K/AKT and MAPK/ERK signaling pathways (*PIK3CA, TEK, GLMN*) have been identified in VM lesion tissue and shown to promote VM pathogenesis (1, 8–17). Under physiological conditions, these two pathways regulate endothelial cell (EC) growth and differentiation. In ECs from VMs, PI3K hyperactivation has been shown to increase cellular proliferation, migration, elongation and cell piling *in vitro*, while ERK pathway activation has been shown to promote cell migration and cell piling *in vitro*. When hyperactivate in venous malformation endothelial cells (VMECs), both the PI3K/AKT and MAPK/ERK the pathways drive increased EC proliferation (3, 15, 18–21).

Venous malformation endothelial cells (VMECs) have been successfully isolated from VMs using antibodies against the EC marker, CD31. These VM-derived ECs were found to have pathogenic variants in both *TEK* and *PIK3CA*, and recapitulated VM phenotype in a xenograft model, demonstrating their pathogenicity (22, 23). We recently demonstrated that the VM endothelium in patient tissues expressed the endothelial progenitor marker CD133, cKIT and CD146 suggesting pathogenic ECs from VMs are immature and failed to terminally differentiate (3). Consistent with this, the VM endothelium also miss-expressed several pan-, venous- and arterial proteins of mature ECs.

Here, we isolated CD133+ cells from VM specimens, characterized their EC gene/protein expression, AKT and ERK activity, and growth rate, performed whole exome sequencing to identify causitive variants in VM genes and assessed their ability to recapitulate the VM patient phenotype in a murine xenograft model. Although heterogenous, CD133+ VM cells misexpressed pan, venous and arterial markers of mature ECs, while displaying increased expression of EC progenitor proteins relative to control ECs. CD133+ VM cells also had pathogenic variants in VM genes *PIK3CA* and *TEK*, and phenocopied the patient VM morphology in a murine xenograft model. Thus, we propose that CD133+ VM cells are a unique population of VMECs that contribute to the development of VMs.

## Materials & Methods

### Human subjects

Resected VM specimens, controls tissues, and chylothorax samples were collected with Columbia University IRB approval (#AAAA9976). The clinical charts were reviewed for VM patient demographics including age, gender, race, ethnicity, and available clinical genetic testing results (Table 1). VMs 7, 9, 24, 33 and 38 were collected prior to the incorporation of genetic testing at our hospital, while VM39 was genetically tested prenatally and postnatally.

**Table 1:** WES and variant analysis of CD133+ and CD133- VM cells.

Cell Population	Age/Sex	Source	Gene	Pathogenic/Likely Pathogenic	Variants of Uncertain Significance (VOUS)	Variant Allele Frequency
CD133+						
7	27, F, His White	Skin/subcutaneous tissue	*PIK3CA*	c.1035T > A p.N345K		0.49
9	12 F, non-His >1 race	Intramuscular	*PIK3CA*	c.1642G > A p.E542K		0.38
24	6F, His Black	Skin/subcutaneous tissue	*PIK3CA*		c.2422C > T p.R808W	0.5
33	3F, non-His Asian	Skin/subcutaneous tissue	*TEK*	c.2740C > T p.L914F		0.495
CD133-						
38	15M, non-His Asian	Skin/subcutaneous tissue	*PIK3R3*		c.1093C > T p.R365*	0.5
39	0 M, non-His White	Chylothorax	*GLMN*	c.108C > A p.C36*		0.5

F, female; M, male; His, Hispanic; non-His, non-Hispanic.

### Cell isolation

CD133+ cell isolation was performed on resected VM specimens or chylothorax fluids as previously described (24–26). Briefly, tissues were digested with collagenase and cell suspension from tissue or fluid was incubated with CD133 antibody conjugated magnetic beads (Miltenyi Biotec) (Supplementary Table 1). CD133+ and CD133- populations of VM cells were seeded on fibronectin-coated tissue culture plates in EC growth media (EBM-2, Lonza), growth factors (EGM-2 Endothelial Growth Medium Bulletkit, Lonza, supplemented with 15% fetal bovine serum), and 1% penicillin and streptomycin (Invitrogen). Cells were characterized by fluorescence-activated cell sorting (FACS) and quantitative reverse transcription polymerase chain reaction (qRT-PCR) to confirm endothelial identity. Two distinct batches of human neonatal microvascular ECs (HMVECs: ATCC, PCS-110-010) and Lonza (#CC-2516) served as controls.

### Fluorescence-activated cell sorting (FACS)

Cell surface protein expression of CD31, CD34, CD45, CD90, CD146, VECADHERIN, VEGFR2, and VEGFR3, were assessed by FACS. 1 × 10^5^ disaggregated cells were incubated with either FITC- or PE-conjugated specific antibody (Supplementary Table 1). As a control, cells were incubated with FITC-IgG or PE-IgG non-specific antibodies. FACS analysis was performed using FACSCalibur and CellQuestPro Acquisition software (BD Biosciences) and analyzed by FlowJo software. After a Ficoll gradient was done to remove red blood cells, 1 × 10^5^ white blood cells from a chylothorax sample were used to validate CD45 FACS.

### IF cell staining of cells

IF staining of cultured cells has been previously described (26–28). Briefly, 100,000 cells per well were seeded onto 4-well chamber slides (Millipore cat. # PEZGS0396) coated with fibronectin. 24-48 h after seeding, cells were fixed with 4% paraformaldehyde (PFA) in phosphate-buffered saline (PBS) on ice for 15 min. After washing in 1X PBS, cells were incubated with primary antibody overnight at 4°C, washed with 1 X PBS followed by secondary antibody for 1 h at room temperature (Supplementary Table 1). After washing in 1 X PBS, slides were mounted with aqueous mounting medium with DAPI (Vector Labs H1200-10). Images were captured using a Zeiss AxioCam MRc camera with Zeiss Zen software at 20X or 40X magnification, an Olympus IX83 Inverted System Microscope, together with the OlympusCellSens software program, or the EVOS M5000 Imaging System at 20X magnification using non-phase lens. All immunofluorescent images were adjusted for contrast and color balance in an identical fashion.

### Quantitative reverse transcription-polymerase chain reaction (qRT-PCR)

RNA was isolated using the RNeasy Mini Kit (Qiagen 74124). cDNA was synthesized using the SuperScript First-Strand Synthesis System (Invitrogen 11904018) (29). Quantitative RT-PCR using gene specific primers (Supplementary Table 2) and Sybr Green Master Mix (Thermofisher) was performed on a CFX96 PCR Cycler (Bio-Rad). A standard curve was generated using serially diluted plasmids containing gene-specific PCR products to determine transcript concentrations which were normalized to β*-ACTIN* levels. Results were obtained from two independently generated cDNAs and qPCR of cDNAs was repeated 2 or more times each.

### Whole exome sequencing (WES), variant calling, classification and validation

Genomic DNA was isolated from CD133+ and CD133- cells using DNAeasy Blood and Tissue kit (Qiagen). The IDT xGen Exome Research Panel V1.0 kit was used to generate a library and exome sequencing (ES) was conducted on the Illumina NovaSeq platform at the Institute for Genomic Medicine (IGM) Columbia University or the New York Genome Center. All samples were processed using a standard IGM bioinformatics pipeline: alignment (Illumina DRAGEN Bio-IT Platform v.2.5.1, hg19), variant calling [Genome Analysis Toolkit (GATK) v3.6], annotation and filtering [in-house Analysis Tool for Annotated Variants (ATAV)] (30). Using the ATAV diagnostic pipeline, we utilized our well-established diagnostic analysis framework (31, 32) to identify clinically significant SNVs and indels that contribute to the VMs phenotype (https://github.com/nickzren/atav/wiki/Diagnostic-Workflow). Each qualifying variant was assessed in the context of the American College of Medical Genetics and Genomics (ACMG) guidelines for variant classification (33, 34). Sequencing results of pathogenic and variants of uncertain significance in genes associated with VMs underwent additional visual inspection of the peaks, and once validated genomic DNAs were sent to Azenta Life Sciences for Sanger sequencing for variant confirmation.

### Western blotting

Total protein was extracted with RIPA Buffer (Thermo Scientific, #89900) supplemented with protease (Thermo Scientific, #78430) and phosphatase (Thermo Scientific, #1862495) inhibitors. Protein concentrations were determined with the Pierce Rapid Gold BCA Protein Assay Kit (A53226, Thermo Scientific) and measured using the ThermoFisher Multiskan Skyhigh Microplate Spectrophotometer. Equal amounts of protein were separated on a 10% SDS/PAGE gel, transferred to nitrocellulose (Bio-Rad, #162-0115), protein transfer visualized by ponceaus S staining, and blocked for 1 h at room temperature in 0.1M Tris-HCl pH 7.5, 150 mM NaCl, 0.1% Tween-20 (1X TBST) containing 4% bovine serum albumin (BSA). Membranes were incubated overnight at 4 °C with primary antibodies at a 1:1,000 dilution in 1X TBST with 4% BSA (Supplementary Table 1). Membranes were washed with 1X TBST and incubated with HRP-conjugated secondary antibodies diluted 1:3,000 in 1X TBST with 4% BSA for one hour at room temperature. Secondary antibodies were detected with SuperSignal West Pico PLUS Chemiluminescent Substrate (Thermo Scientific, #34577) and the iBright 1500 Imaging System (Invitrogen). Band intensities for six sets of lysates were quantified using ImageJ software, normalized to control HMVECs.

### Proliferation assay

40,000 HMVECs (*n* = 2 populations) or CD133- cells were seeded in one well of 6-well plate and cell number determined after 24 h. Cells used ranged from passage 7-11. Cell numbers was determined using hemacytometer and averaging 16 quadrants. Cell number for three independent wells per cell type were normalized to average HMVEC proliferation rate and presented fold of HMVEC control.

### Xenograft model

All animal work was approved by Columbia University's IACUC (AAAQ8400, AABC7500, AABS6605). A total of 1.5 × 10^6^ cells derived from VMs were resuspended in 200*μ*L Matrigel (Corning, 356237) and injected subcutaneously into each flank of NCr female mice (Taconic). After 4 weeks, Matrigel implants were harvested, fixed in 4% paraformaldehyde and paraffin-embedded for histological and immunohistochemical analyses (26, 35).

### Immunostaining of sections

Five micron sections of frozen or paraffin-embedded tissues or implants were generated. Patient VM tissues were hematoxylin and eosin (H&E) stained by Columbia University's Molecular Pathology Shared Resources Core.

For colorimetric staining, paraffin sections were deparaffinized, rehydrated, and then endogenous avidin and biotin blocked (Avidin/Biotin Blocking Kit, Vector Laboratories). Sections were blocked in 3% BSA in 1 X PBS, and incubated with an antibody against human VECADHERIN in blocking solution overnight at 4°C (Supplementary Table 1) (36). Primary antibody was detected using a biotinylated rabbit anti-goat IgG antibody (Vector Laboratories BA-5000), incubated at room temperature for 30 min followed by detection with Vectastain Elite ABC-HRP Kit (PK6100, Vector Laboratories) and DAB substrate (SK-5100, Vector Laboratories). Slides were counterstained with hematoxylin, mounted with permount.

For IF staining of paraffin sections, tissues were deparaffinized, and rehydrated. For IF staining of frozen sections, slides were fixed in cold acetone. Both paraffin and frozen sections were then blocked in 5% BSA in 0.1% TX-100, 1 X PBS for an hour at room temperature. Sections were incubated at 4 °C overnight in primary antibody (Supplementary Table 1) diluted in blocking solutions. After washing in cold 1 X PBS, sections were incubated in blocking solution containing flourscent secondary antibody for 1 h a room temperature. Sections were washed in cold 1 X PBS and mounted with aqueous mounting medium with DAPI.

Slides were imaged using a Zeiss AxioCam MRc camera with Zeiss Zen software (20X or 40X magnification), or a Olympus IX83 Inverted System Microscope, the OlympusCellSens software program, or the EVOS M5000 Imaging System at 20X magnification using non-phase lens. All images of stained tissues immunostain were adjusted for contrast and color balance in an identical fashion.

### Statistical analysis

One-way ANOVA with *post-hoc* Tukey *T-Test* was used for statistical analysis using Graphpad Prism v10, and a *p* value of <0.05 was considered statistically significant.

## Results

### Isolation of a CD133+ endothelial cell population from VM specimens

We previously found that the VM endothelium was improperly specified with altered expression of pan-EC genes, CD31, VECADHERIN and VEGFR2, reduced expression of the venous EC marker, COUPTF-II and increased expression of DLL4 which was associated with an increase vascular smooth muscle cell coverage suggesting the VM endothelium failed to properly specify (3). Consistent with this hypothesis, the VM endothelium expressed the EC progenitor cell markers, CD133, cKIT and CD146. As a CD133 stem/progenitor cell population in infantile hemangioma and lymphatic malformations have been shown to recapitulate their vascular phenotypes, we aimed to isolated the CD133+ cell population from VM specimens and assess their phenotype. To study these cells further, CD133+ and CD133- cells were isolated from 6 VM specimens. Four of the 6 VM specimens were located in the skin and subcutaneous tissues. One VM specimen was located intra-muscularly, and the last specimen was collected from congenital chylothorax fluid (Table 1). CD133+ cells were successfully isolated from 4 VMs, while CD133- cells were isolated from 5 VMs, with matched CD133+/CD133- cells isolated for 3 VMs.

**Figure 1:**
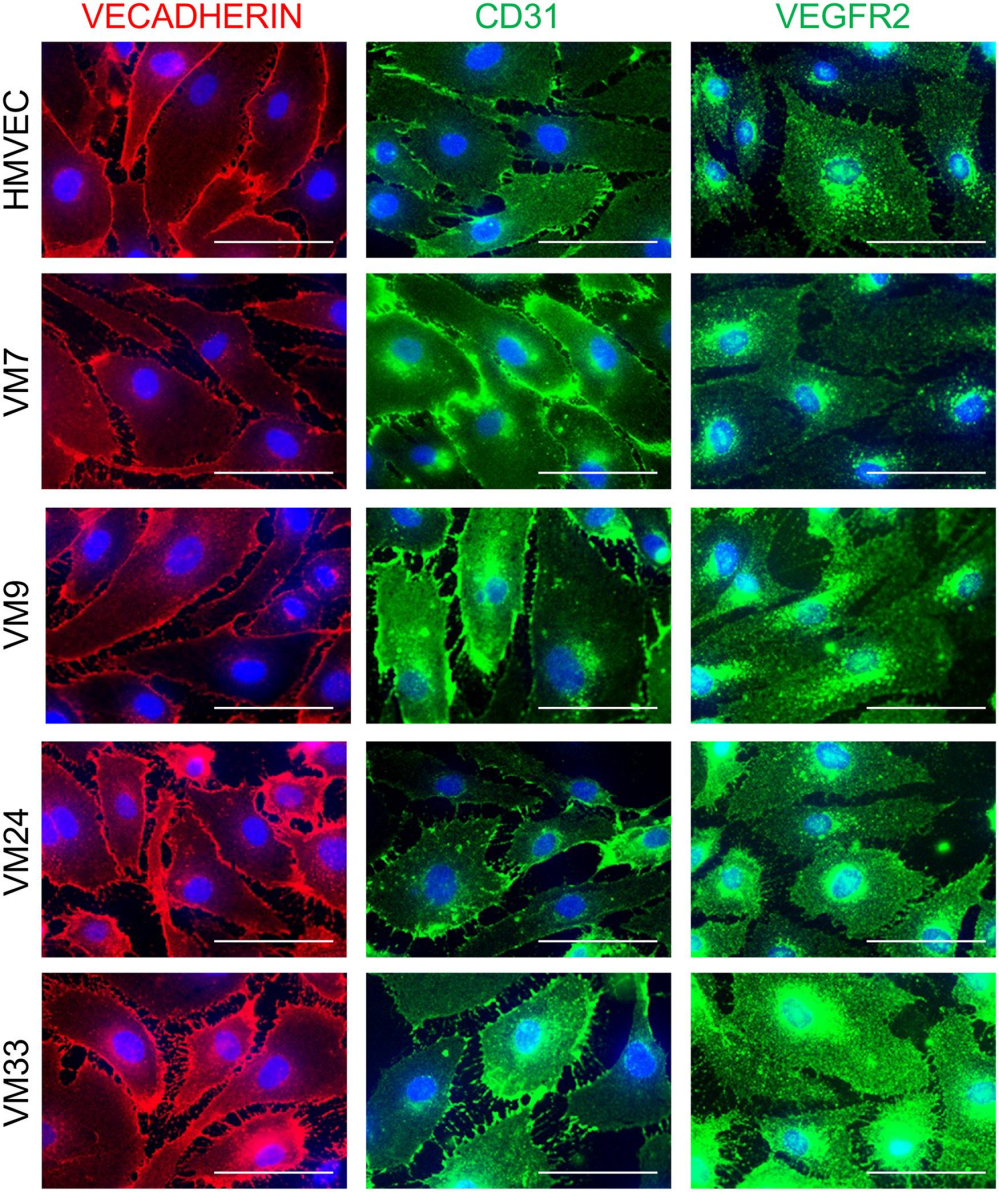
VMECs express endothelial proteins. HMVECs and CD133+ VM cells stained for VECADHERIN, CD31, and VEGFR2. Scale bars 50 μm. ECs, endothelial cells; HMVECs, human microvascular endothelial cells; VMECs, venous malformation endothelial cells.

To characterize CD133+ VM cell populations, IF staining was done to assess expression of pan-EC proteins, CD31, VECADHERIN, and VEGFR2 in 4 CD133+ VM cell popupations. Similar to control HMVECs, CD133+ VM cells expressed CD31, VECADHERIN, and VEGFR2 (Figure 1).

**Figure 2:**
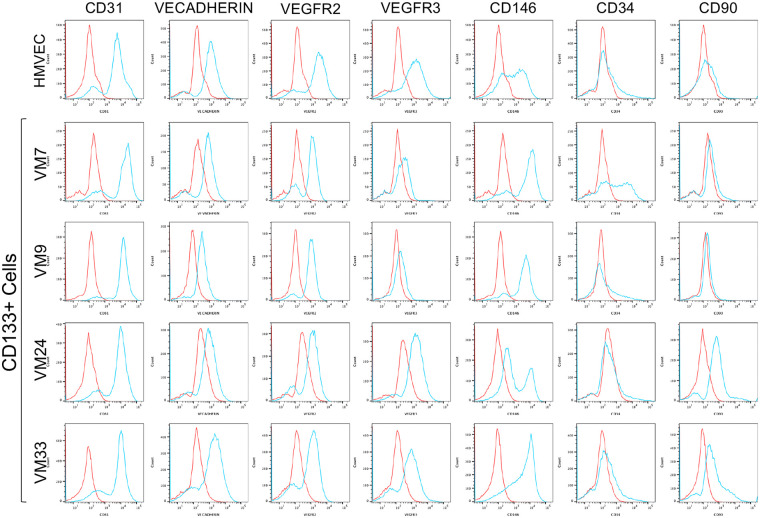
Expression of endothelial proteins at the surface of CD133+ cells. FACS analysis of CD31, VECADHERIN, VEGFR2, VEGFR3, CD146, CD34, and CD90 in HMVECs and CD133+ VM cells. Blue lines represent FACs with specific antibodies. Red lines represent IgG controls. HMVEC, human dermal microvascular endothelial cells; VM, venous malformation.

Further cell surface expression analysis was done by FACS. In addition to CD31, VECADHERIN, and VEGFR2, we evaluated VEGFR3, which is expressed in both venous and lymphatic ECs (37–41) and has been shown to be expressed in VMs (42). To assess expression of endothelial progenitor proteins, FACs was done with antibodies against CD146 (43) and CD34 (44, 45), as well as CD90, which is expressed on activated blood ECs (46) and lymphatic ECs (47). CD45 expression was assessed to ensure the isolated cells were not immune cells which can also express CD90, and CD146 (48). All CD133+ VM cells expressed CD31, VEGFR2 and CD146 at the cell surface similar to HMVECs (Figure 2). CD133+ cells expressed VECADHERIN and VEGFR3, but their expression at the surface relative to HMVEC was reduced in 1/4 and 2/4 populations, respectively. Expression of EC progenitor markers at the surface was heterogenous in CD133+ cells. CD34 was not expressed in 3/4 CD133+ cells and HMVECs, while CD90 expression was increased in 3/4 CD133+ populations relative to HMVECs. Together, this data demonstrate that the CD133+ cells derived from VMs express multiple EC proteins and progenitor cell markers, but with altered expression and localization relative to control ECs (Figure 2).

FACS of CD133- VM cells revealed that they also expressed EC markers, but their expression was often decreased at the cell surface relative to HMVECs. 4/5 CD133- populations expressed CD31 and VEGFR2 similar to HMVECs. In contrast, VECADHERIN surface expression was down in 4/5 CD133- populations relative to HMVEC, while VEGFR3 expression was reduced in 3/5 populations. CD133- cell populations sometimes had higher expression of endothelial progenitor cell markers. The majority (4/5) of CD133- cells expressed CD146 similar to HMVECs, while 3/5 CD133- populations had increased CD34. CD90 expression was low or absent in most CD133- populations. One CD133- population (VM39) with increased CD90 expression relative to HMVEC had the lowest surface expression of EC markers with only low expression of VEGFR3 and CD146 (Supplementary Figure 1). FACs for CD45 confirmed the isolated cells were not immune cells (Supplementary Figure 2).

Taken together, these data showed that both CD133+ and CD133- cells isolated from VMs express pan-EC proteins but often had reduced surface expression of one or more them, while some had increased progenitor cell proteins suggesting failure to terminally differentiate similar to what was observed in VM patient tissues (3).

### CD133+ and CD133- cells have variants in VM genes

To determine if the CD133+ or CD133- VM cells contain pathogenic variants, we subjected them to WES and variant characterization. By performing WES on the enriched cells, we aimed to capture both germline and somatic variants in genes associated with VMs (49). VM9-CD133+ cells had a pathogenic *PIK3CA^E542K^* variant, and VM33-CD133+ cells had a pathogenic *TEK^L914F^* variant, both of which have both previously been reported in VMs (Table 1). VM7-CD133+ cells had a *PIK3CA^N345K^* variant that has yet to be reported in VMs, but has been shown to be pathogenic in cancers (50, 51). Variants of unknown significance (VUS) were detected in *PIK3CA* (*PIK3CA^R808W^*) and *PIK3R3* (PIK3R3^R365*^), in VM24 and VM38 cells respectively. Finally, VM39 CD133- cells had a likely-pathogenic variant in *GLMN* (GLMN^C36*^) which was confirmed to be germline when assessed via clinical genetic panel. Prenatal diagnosis of congenital chylothorax that subsequently manifests with plaque-like venous malformations has been reported (52, 53). Due to limited clinical data and the high variant frequency observed we are unable to confirmed whether the variant were somatic in most CD133+ VM cells, except for VM9 CD133+ cells that had a VAF of 38% consistent with this population being enriched for cells with a somatic *PIK3CA^E542K^* variant and previously observed in LM derived cells (54). Sanger sequencing confirmed all variants were present. These genetic findings suggest that both CD133+ and CD133- VM cells are improperly specified ECs have causative genetic variants or VUS, which we termed CD133+ VMECs and CD133- VMECs (Supplementary Table 3).

### Gene expression analysis of CD133+ VMECs with PIK3CA and TEK variants

We further characterized the EC gene expression patterns of CD133+ VMECs with the *PIK3CA* and *TEK* variants, as these genes are commonly mutated in VMs. Quantitative RT-PCR was performed for the pan-EC (*CDH5*, *FLT1*, *KDR*, *ANGPT2*), and venous EC (*FLT4*, *COUP-TFII*, *EPHB4*) genes. As we observed an increased in endothelial DLL4 expression and vascular smooth muscle cell coverage around VM vessels in patient tissues (3), we also analyzed the arterial EC genes (*DLL4*, *EPHRINB2*) and *ANGPT1* as it can be expressed by ECs and is involved in vascular smooth muscle recruitment (55, 56).

**Figure 3:**
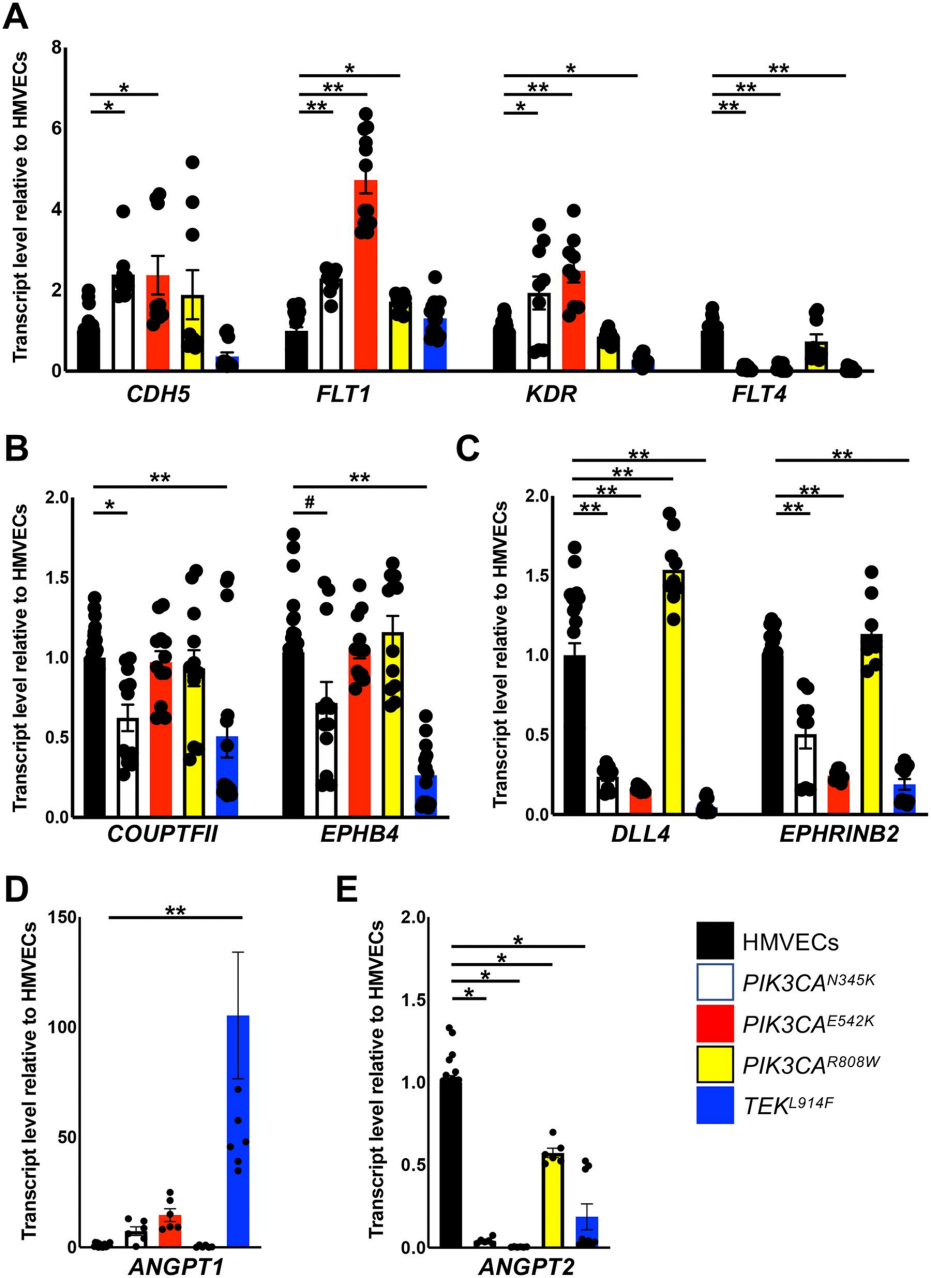
CD133+ VMECs with *PIK3CA* and *TEK* variants differentially misexpressed EC genes. qRT-PCR of CD133+ VMECs and HMVECs for **(A)** BEC genes; *CDH5, FLT1, KDR, and FLT4* **(B)** venous-EC genes; *COUPTFII* and *EPHB4,* **(C)** arterial-EC genes; *DLL4* and *EPHRINB2*, **(D)** *ANGPT1* and **(E)** *ANGPT2*. qRT-PCRs were performed in triplicate, and data for ≥3 biological replicates presented as fold expression normalized to HMVECs ± sem. ANOVA p < 0.0001. *Post-hoc* Tukey tests: #*p* < 0.05, $*p* < 0.01, **p < 0.0001, *p < 0.005. HMVECs, human microvascular endothelial cells; BEC, blood endothelial cells.

Gene expression was variable between the different CD133+ VMEC populations relative to control HMVECs (Figure 3). In CD133+ VMECs with *PIK3CA^N345K^* and *PIK3CA^E542K^* variants, a significant increase in *CDH5*, *FLT1* and *KDR*, and decrease in *ANGPT2, DLL4,* and *EPHRINB2* transcripts was observed relative to HMVECs. Reduced expression of venous EC genes, *FLT4*, *COUPTFII* and *EPHB4* was also observed in *PIK3CA^N345K^* CD133+ VMECs. *PIK3CA^R808W^*CD133+ VMECs gene expression pattern was distinct from the other *PIK3CA* variants with an increase in *FLT1* and *DLL4* transcripts and a modest decrease in *ANGPT2* expression relative to HMVECs. *TEK^L914F^* CD133+ VMECs had decreased expression of most genes assessed except for *ANGPT1* which was significantly increased relative to HMVECs. Together with the FACS data, these results indicate that CD133+ VMECs isolated from patient VMs expressed EC proteins and gene transcripts, but their expression patterns differ between normal HMVECs and each other.

### Subset of CD133+ VMECs had increased nuclear AKT activation

**Figure 4:**
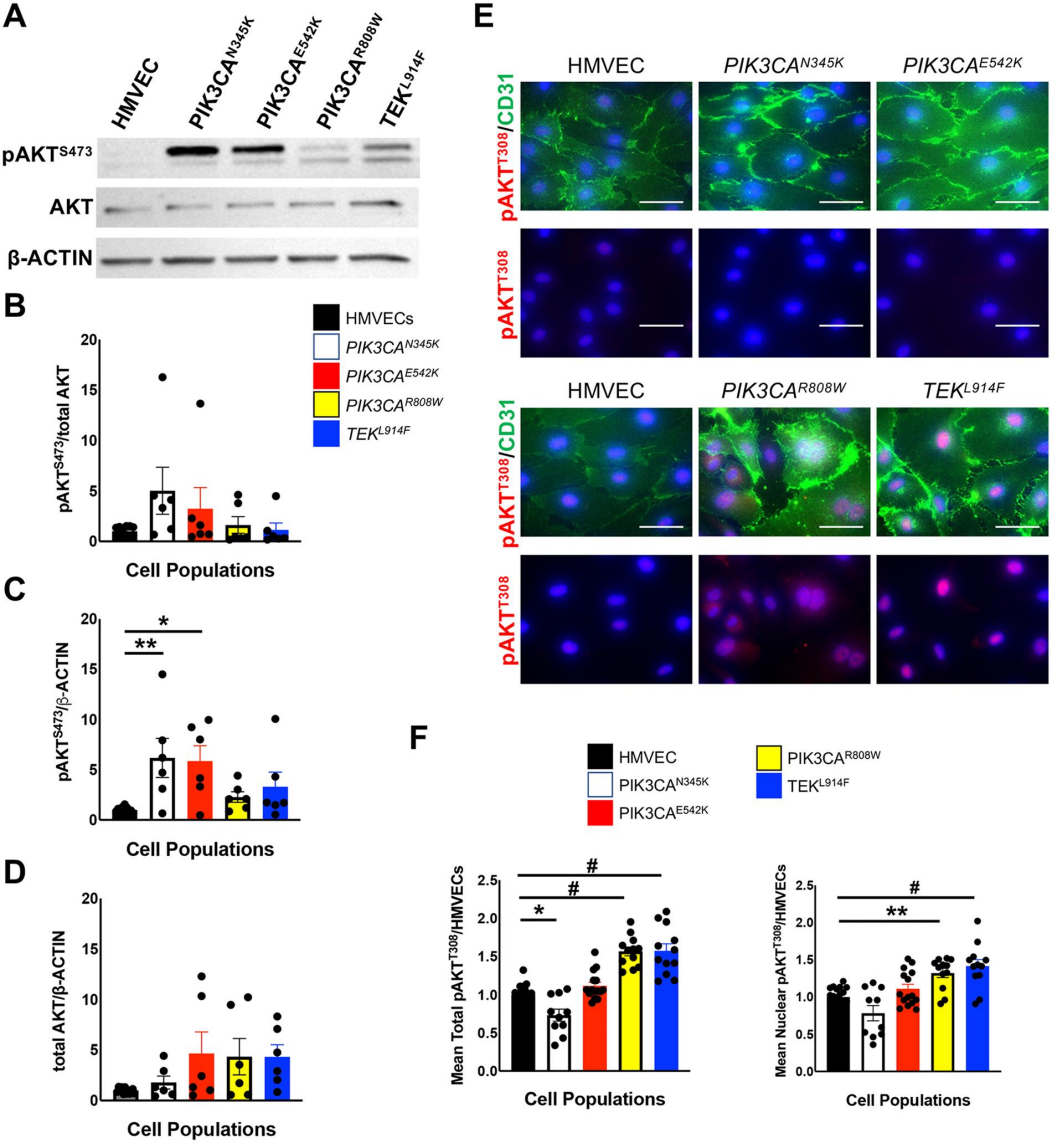
Baseline AKT activation in CD133+ VMECs with *PIK3CA* and *TEK* variants. **(A)** Representative Western blot for pAKT^S473^, total AKT, and β-ACTIN. Mean **(B)** pAKT^S473^/total AKT, **(C)** pAKT^S473^/β-ACTIN and **(D)** total AKT/β-ACTIN expression. Data presented for 6 experiments normalized to HMVECs ± sem. ANOVA, *p* < 0.005, *post-hoc* Tukey test *p < 0.05, **p < 0.01. (**E)** pAKT^T308^ and CD31 staining of HMVECs and VMECs. Scale bars 50 μm. Mean total (**F)** pAKT^T308^ and nuclear pAKT^T308^ signal intensity normalized by number of HMVEC. Data presented for two experiments ± sem. ANOVA *p* < 0.0001. *Post-hoc* Tukey test *p < 0.05, **p < 0.01, #*p* < 0.0005. HMVECs, human microvascular endothelial cells; pAKT, phosphorylated AKT; VMECs, venous malformation endothelial cells.

As *PIK3CA* and *TEK* variants in VMs are predicted to activate PI3K/AKT/mTOR and MAPK signaling (15, 57, 58), AKT and ERK activation in CD133+ VMECs and HMVECs were assessed under normal growth conditions. AKT activation was determined by Western blot using an antibody against AKT phosphorylated at S473 (pAKT^S473^) and by IF using an antibodies against AKT phosphorylated at T308 (pAKT^T308^) or S473 (pAKT^S473^) (Figure 4). When assessed by Western blot, pAKT^S473^ normalized to total AKT did not significantly differ between the CD133+ VMECs and HMVECs, though cells with *PIK3CA^N345K^* and *PIK3CA^E542K^* variants were trending higher (Figures 4A,B). When pAKT^S473^ was normalized to *β*-ACTIN, pAKT ^S473^ levels were increased significantly in CD133+ VMECs with *PIK3CA^E542K^* and *PIK3CA^N345K^* variants (Figures 4A,C). This difference may be due to an increase in total AKT in CD133+ VMECs, as total AKT normalized to β-ACTIN, trended higher compared to HMVECs (Figures 4A,D).

IF staining using antibodies against either AKT phosphorylation site, S743 or T308, were significantly increased in the nucleus of CD133+ VMECs with *PIK3CA^R808W^* and *TEKL^914F^* variants (Figures 4E,F; Supplementary Figure 3). As there was no difference in pAKT^S473^ normalized to total AKT these cells detected by Western, our IF results suggest that in some pathogenic CD133+ VMECs AKT signaling activates distinct proteins in the nucleus than those at the cell surface in normal ECs.

**Figure 5:**
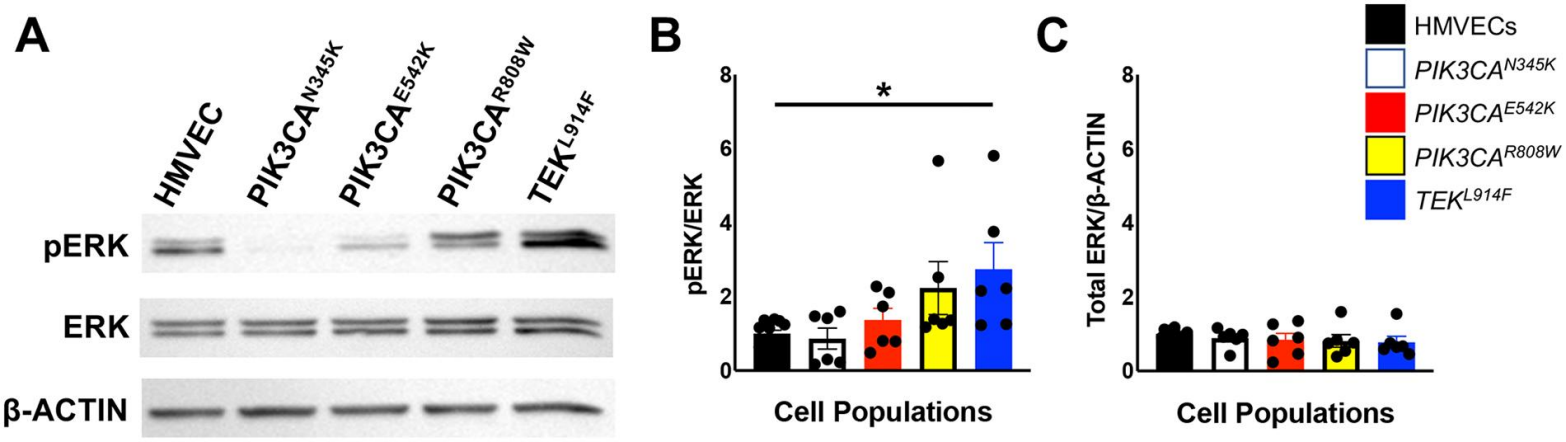
Baseline ERK activation in CD133+ VMECs with *PIK3CA* and *TEK* variants. **(A)** Representative Western blots for pERK, total ERK, and β-ACTIN. Mean **(B)** pERK/total ERK, and **(C)** total ERK/β-ACTIN expression. Data presented for 6 experiments normalized to HMVECs ± sem. ANOVA, *p* < 0.02. *Post-hoc* Tukey *t*-test *p < 0.05. HMVECs, human dermal microvascular endothelial cells.

MAPK pathway hyperactivation was assessed by Western blot using antibodies against phospho-ERK (pERK) and ERK. Only CD133+ VMECs with the *TEK*^L914F^ variant had significantly higher levels of pERK normalized to total ERK relative to HMVECs (Figure 5). Thus, in growth conditions only CD133+ VMECs with the *TEK* variant had increased MAPK signaling, as well as nuclear AKT signaling.

### CD133+ VMECs had increased proliferation

**Figure 6:**
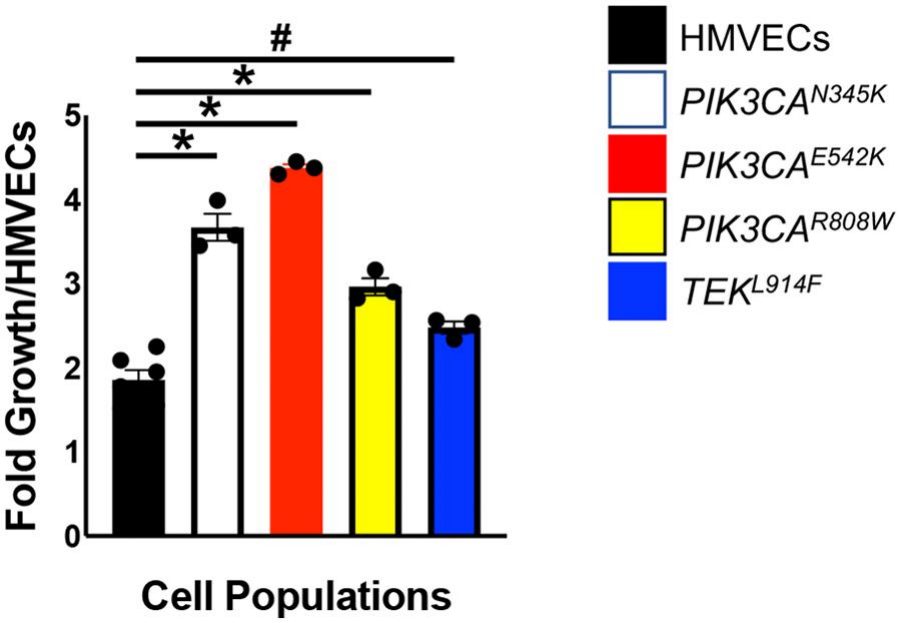
Assessment of CD133+ VMECs with *PIK3CA* and *TEK* variants growth at 24 h. Data for experiments with ≥3 biological replicates presented as fold growth normalized to HMVECs ± SEM. ANOVA, *p* < 0.0001. *Post-hoc* Tukey *p ≤ 0.0001, #*p* < 0.02. HMVECs, human microvascular endothelial cells.

As *PIK3CA* and *TEK* variants have been associated with increased proliferation in engineered ECs (42, 59–62), CD133+ VMECs and HMVEC (*n* = 2 populations) growth rates in complete media were determined. After 24 h, all CD133+ VMECs grew significantly faster than control HMVEC in normal growth conditions (Figure 6).

### CD133+ VMECs recapitulated VM phenotype in a murine xenograft mode

**Figure 7:**
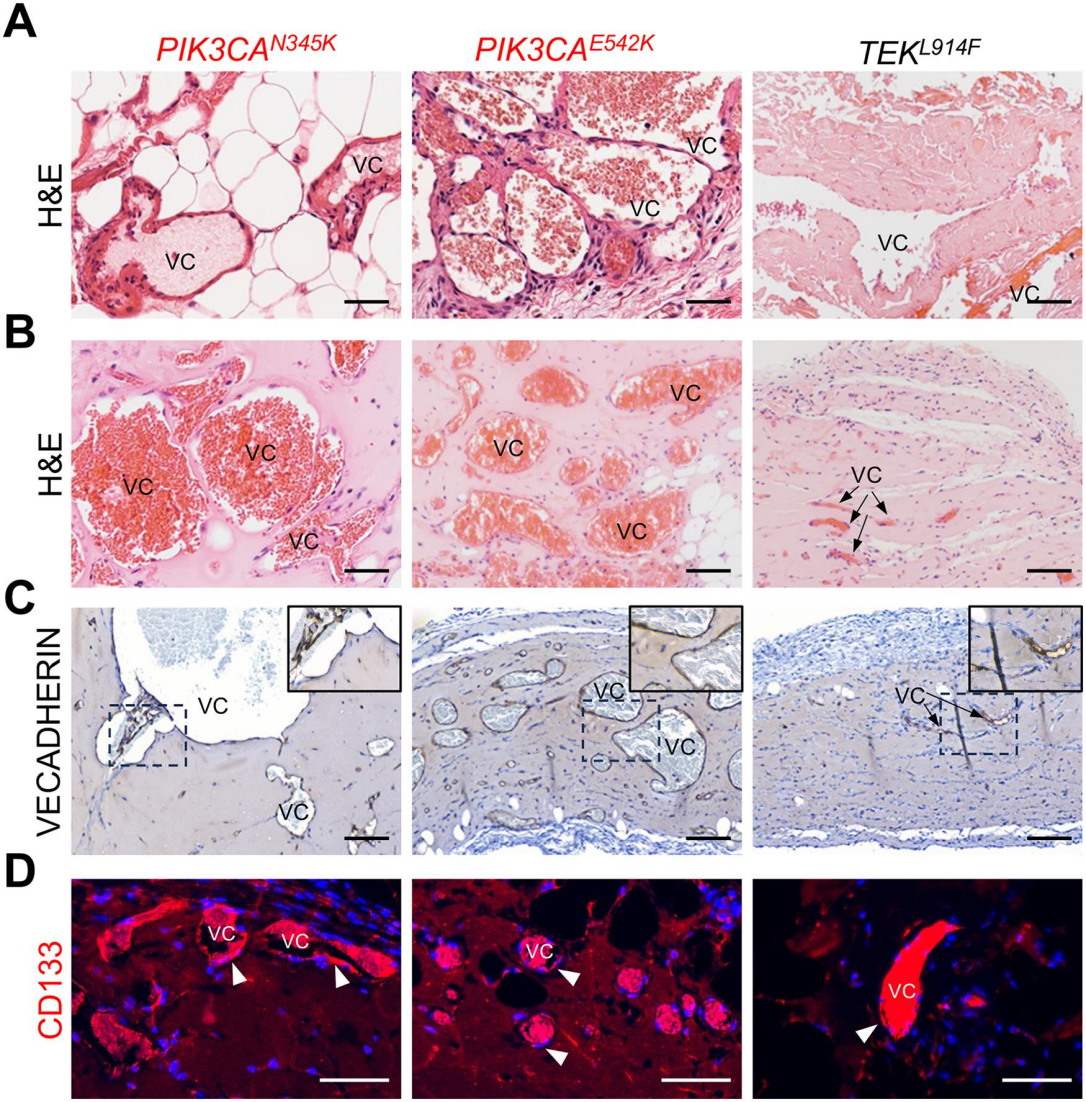
Xenografts of CD133+ VMECs with *PIK3CA* and *TEK* variants phenocopied clinical histology. **(A)** H&E staining of patient VM tissues with *PIK3CA^N345K^*, *PIK3CA^E542K^* or *TEK^L914F^* variants. **(****B)** CD133+ VMECs xenograft implants stained for H&E, **(C)** VECADHERIN, and **(D**) CD133. Vascular channels labeled with “VC” inside the VM vessel lumen or marked with arrows. White arrowheads mark CD133+ cells. **(C)** Dotted boxes are enlarged in the upper right corner of images. **(A–C)** Scale bars 100 μm, **(D)** 50 μm. *n* ≥ 4 xenografts per CD133+ VMEC population were done in triplicate for *PIK3CA^N345K^*, *PIK3CA^E542K^* or duplicate for *TEK^L914F^*.

In order to assess if CD133+ VMECs can phenocopy patient VMs in a xenograft mouse model, CD133+ VMEC populations were resuspended in Matrigel, and implanted subcutaneously in immunocompromised mice. After 4 weeks, the implants were removed, and paraffin sections stained with H&E and a human-specific EC antibody, VECADHERIN (Supplementary Figure 4). Xenograft implants were compared to matching clinical specimens (Figure 7).

*PIK3CA^E542K^*, *PIK3CA^N345^* and *TEK^L914F^* CD133+ VMEC implants displayed large dilated, disorganized and blood-filled vascular channels (Figure 7B), similar to those observed in the patient-matched tissues (Figure 7A). CD133+ VMEC with *PIK3CA* variants had enlarged and round vascular channels similar to those observed in the patient tissues. *TEK^L914F^* CD133+ VMECs developed elongated dilated channels that were similar to some vessels in the matching patient tissues. VECADHERIN expression detected with a human-specific antibody was observed in the cells lining the dilated vascular channels, confirming that the endothelium arose from the human CD133+ VMECs rather than endogenous murine ECs (Figure 7C; Supplementary Figure 4). Mice injected with normal HMVECs did not develop enlarged vascular channels (Supplementary Figure 5). Next, we determined if CD133 expression was maintained. Immunostaining of *PIK3CA^E542K^*, *PIK3CA^N345^* and *TEK^L914F^* CD133+ VMEC implants, revealed spotty endothelial CD133 expression in the enlarged VM vessels similar to that observed in VM specimens (Figure 7D; Supplementary Figure 6) (3).

**Figure 8:**
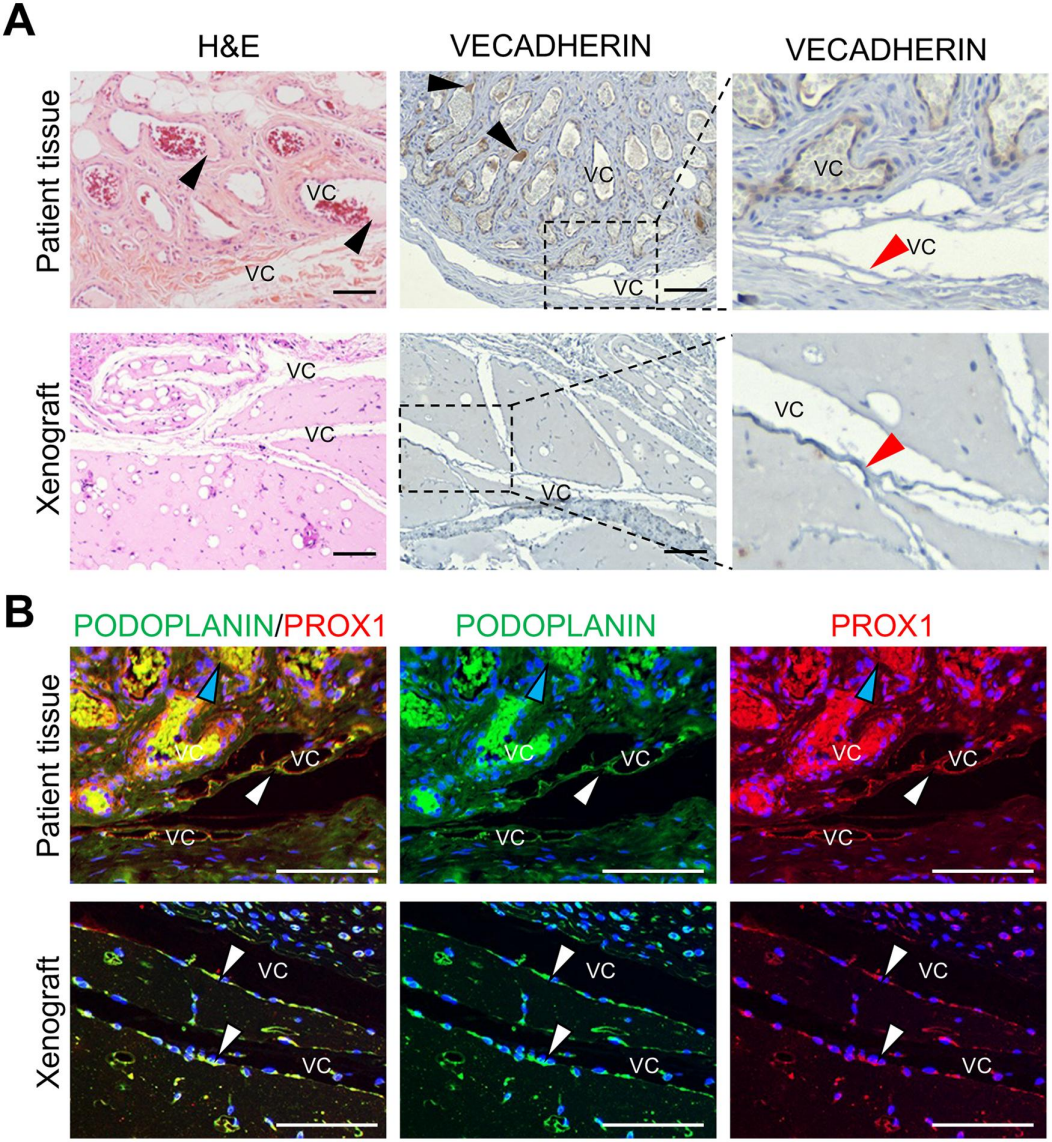
Xenografts of *PIK3CA^R808W^* CD133+ VMECs with *PIK3CA^R808W^* variants revealed a lymphatic endothelial cell component. **(A)** H&E and VECADHERIN staining of *PIK3CA^R808W^* patient VM tissue and CD133+ VMEC xenograft. Black arrowheads mark proteinaceous fluid consistent with lymph in VCs. Red arrowheads highlight sloughing endothelium. Boxed areas are enlarged to the right. **(B)** PODOPLANIN and PROX1 staining of *PIK3CA^R808W^* patient VM tissue and CD133+ VMEC xenograft. White arrowheads mark PODOPLANIN+/PROX1 + cells, and blue arrowheads mark PODOPLANIN-/PROX1- cells. Scale bars 100 μm. *n* ≥ 4 xenografts were done in triplicate. Pt, patient; VC, vascular channel

Implants from *PIK3CA^R808W^* CD133+ VMECs did not form dilated vessels like those observed in the patient-matched VM specimen (Figure 8). Instead *PIK3CA^R808W^* CD133+ VMECs formed elongated channels with the endothelium sloughing into the vascular channels that was either negative or weak for VECADHERIN, which was also observed in the patient VM specimen (Figure 8A). As these elongated channels were similar to those observed in implants with ECs from lymphatic malformations (LMs) (26), and proteinaceous fluid consistent with lymph was observed in the dilated vessels, xenograft and patient tissues were stained for lymphatic endothelial cell markers, PODOPLANIN and PROX1. Both the clinical specimen and xenograft vascular channels were lined by PODOPLANIN+/PROX1 + endothelial cells like lymphatics in normal neonatal skin (Figure 8B; Supplementary Figure 7). PROX1, a transcription factor, was observed in the cytoplasm and not the nucleus similar to what has been described for LM vessels (63). Unlike the dilated VM vessels, the endothelium of *PIK3CA^R808W^* CD133+ VMEC did not express CD133 (data not shown). Together these data suggests that the *PIK3CA^R808W^* variant is pathogenic in CD133+ VMEC.

As we observed PODOPLANIN expressed by *PIK3CA^R808W^* CD133+ VMEC xenografts, and VM39 CD133-VMECs were isolated from chylothorax fluid (Table 1), we assessed PODOPLANIN expression by FACS analysis. CD133- VMECs from VM39 from a GVM case expressed PODOPLANIN on its cell surface (Supplementary Figure 8). Taken together VM39 CD133- VMECs were PODOPLANIN positive, VEGFR3 low and CD146 low suggesting they have characteristics of lymphatic endothelial cells (LECs). Whether venous lesions in GVM also express PODOPLANIN has not been described.

## Discussion

Characterization of VM patient tissues revealed that the VM endothelium failed to properly differentiation, seen as reduced expression in pan EC proteins, CD31 and VECADHERIN and venous EC protein COUP-TFII, and increased in the arterial EC protein, and vascular smooth mucle cell coverage (VSMCs) (3). The VM endothelium also expressed spotty endothelial progenitor cell marker, CD133, CD146 and cKIT. In LMs and infantile hemangioma, CD133+ cells were found to be progenitor-like in that they were pluripotent and able to recapitulate the vascular anomaly phenotype in murine xenograft models (26, 35). To understand the role of the potentially progenitor-like cell population in VMs, we isolated CD133+ cell populations. Both CD133+/CD133- VM cell populations expressed EC proteins and transcripts, albeit at varied levels relative to control HMVECs, as well as each other, confirming that they are ECs (Table 2). Except for *ANGPT2*, *FLT4/*VEGFR3, *DLL4* and *COUP-TFII*, a majority of the endothelial progenitor, pan-EC, venous EC, and arterial EC genes were not consistently altered relative to HMVECs. Consistent with our findings of reduced *ANGPT2* levels in all CD133+ VMECs*,* recent publications have shown that *PIK3CA* variants in both LMs and VMs leads to decreased EC-derived *ANGPT2/*ANG2 expression (16, 64, 65). In 3/4 CD133+ VMEC populations (*PIK3CA*^N345K^, *PIK3CA*^E542K^, and *TEK*^L914F^) a decrease in *FLT4* transcripts, VEGFR3 at the cell surface, and arterial EC genes, *DLL4* and *EPHRINB2* was observed*.* Relative to the VM9, VM24, and VM33-derived CD133+ VMECs, patient-matched CD133- VMECs had reduced VECADHERIN and CD90 on the cell surface. Additional CD133+ and CD133- VMEC populations will need to be characterized to determine if these changes in genes and protein expression are common to VMs or correlate with specific genetic variants.

**Table 2:** Summary of data for CD133+ VMECs relative to HMVECs

Protein/gene	*PIK3CA* ^N345K^				*PIK3CA* ^E542K^				*PIK3CA* ^R808W^				*TEK* ^L914F^			
	FACS	IF	qRT-PCR	WB	FACS	IF	qRT-PCR	WB	FACS	IF	qRT-PCR	WB	FACS	IF	qRT-PCR	WB
CD31	nc	exp			nc	exp			nc	exp			nc	exp		
VECADHERIN/*CDH5*	−0.5	exp	+2		−0.5	exp	+2		−0.5	exp	nc		nc	exp	nc	
VEGFR1/*FLT1*			+2.5				+4				+2				nc	
VEGFR2/*KDR*	−0.5		+2		−0.5		+3		−0.5		nc		−0.5		−0.5	
VEGFR3/*FLT4*	−0.5		−0.2		−0.5		−0.2		nc		nc		nc		−0.2	
CD146	nc				nc				nc				nc			
CD34	+1				nc				nc				nc			
CD90	+0.5				nc				+1				+0.5			
COUPTFII			−0.5				nc				nc				−0.5	
EPHB4			−0.75				nc				nc				−0.25	
DLL4			−0.25				−0.25				+1.5				−0.1	
EPHRINB2			−0.5				−0.25				nc				−0.25	
*ANGPT1*			nc				nc				nc				+100	
*ANGPT2*			−0.1				−0.1				−0.5				−0.2	
pAKT^S473^		nc		nc		nc		nc		+1.5		nc		+2		nc
pAKT^T308^		nc				nc				+1.5				+1.5		
pERK/ERK				nc			nc					nc				+3

nc, no change; IF, immunofluorescence; WB, western blot; HMVECs, human microvascular dermal endothelial cells. FACS: right (+) or left (−) log shift relative to HMVECs. IF, exp, expressed by immunofluorescence; none, not expressed by immunofluorescence. Alternatively, fold increase was presented relative to control HMVEC (value = 1). qRT-PCR: relative transcript levels increased (+) or decreased (−) relative to control HMVEC (value = 1). WB: relative densitometry levels fold increased (+) or decreased (−) relative to control HMVECs (value = 1); represents total pAKT/total AKT and pERK/total ERK.

Our genetic studies identified both causative variants and VUS in isolated CD133+ VMECs. Two of the CD133+ VMECs had pathogenic variants, *PIK3C^E545K^* and *TEK^L914F^*, previously described in sporadic VMs (12–14, 16, 57, 58, 66). Two *PIK3CA variants, PIK3CA^N345K^* and *PIK3CA^R808W^*, identified in the CD133+ VMECs have not been described in VMs. The *PIK3CA^N345K^* variant is in the C-domain is reported as likely-pathogenic in ClinGen. The *PIK3CA^R808W^* variant is in the kinase domain and listed as VUS in ClinGen related to Cowden's disease and an unspecified inborn genetic disease. It has also been described in neoplasms of the large intestines and breast (67). As *PIK3CA^N345K^* and *PIK3CA^R808W^* CD133+ VMECs have increased proliferation and recapitulate the patient VM phenotypes in xenografts, these data support them being causative in VMs. The *PIK3CA* and *TEK* variants in CD133+ VMECs derived from VM7, VM9, and VM24 had a variant frequency (VAF) between 0.49 and 0.50. As clinical genetic testing was not done on these cases, it is unclear whether these variants are germline or somatic. Although both germline and somatic variants have been described for *TEK* in VMs (49), germline heterozygous activating *PIK3CA* variants are not compatible with life (68) and only somatic *PIK3CA* variants have been described in VMs and LMs. Thus, the *PIK3CA* variants in VM7 and VM9 are most likely somatic. Consistent with this, the VAF of CD133+ VMECs from VM9 was 0.38 suggesting we enriched for VMECs with a somatic pathogenic *PIK3CA* variant that contributes to the development of VMs.

Under growth conditions AKT activation in CD133+ VMECs with *PIK3CA* or *TEK* variants was weak and did not reach significance. In a mouse model of *PIK3CA* driven LMs, blocking macrophage-derived VEGF-C suppressed the development of the malformation (69). Thus, hyperactivating *PIK3CA* or *TEK* variants in the CD133+ VMECs may be dependent on activation of cell surface receptors, such as TIE2. We did observe a significant increase of AKT activation in the nucleus of CD133+ VMECs with the *TEK^L914F^* and *PIK3CA^R808W^* variants. Two recent papers have shown that there is a pathological PIK3CA/FOXO1/ANG2/VEGFR3 signaling cascade in ECs in LMs and VMs (64, 70). In LECs, hyperactivation of PIK3CA led to FOXO1 phosphorylation via AKT, which in turn reduced *ANGPT2* transcription leading to VEGFR3 degradation (64, 71). Kraft et al. showed a similar role for *PIK3CA* variants in VMs in which hyperactivated PIK3CA was shown to inhibit FOXO1 and decrease the TIE2 agonist, *ANGPT2* leading to increased TIE2 activation (70). Thus, the nuclear pAKT in CD133+ VMECs may target FOXO1 for degradation and be responsible for the decrease in *ANGPT2* transcripts we observed. The nuclear pAKT in *TEK^L914F^* and the *PIK3CA^R808W^*CD133+ VMECs was also associated with the presence of elongated vascular channels with sloughing endothelium in both patient tissues and xenografts. In contrast, nuclear pAKT did not correlate with a common gene/protein signature which may be due to our study being insufficiently powered.

Although activation of ERK has been reported in for some ECs with *PIK3CA* variants (65), only CD133+ VMECs with the *TEK* variant had increased phosphorylation of ERK under growth conditions. This activation of ERK in ECs with the *TEK^L914F^* variant is consistent with prior reports (22, 72). Interestingly, TIE2 signaling via the ERK pathway has been shown to promote ANG1 expression in a positive feedback loop in ECS (73). Thus, the increase in *ANGPT1* transcripts observed in *TEK^L914F^* CD133+ VMECs may be due to the increased ERK activity.

A potential limitation to our study is the heterogeneity in the CD133+ VMECs studied. Due to the small number of CD133+ VMEC populations analyzed, expression patterns between populations were variable. Further studies using more cell populations from different patients is needed to determine any correlations between genetic variants and biologic patterns. Furthermore, using CD133 for cell isolation may result in isolation of non-ECs; however, EC identity was confirmed by expression analysis before being used in experiments.

In conclusions, we have isolated and characterized a yet to be described cell type in VMs, the CD133+/CD31+ VMEC, that have pathogenic variants, has increased proliferation, and can recapitulate the VM phenotype in a xenograft model. CD133+ cells have been described to contribute to abnormal vessel development for other vascular anomalies. Hemangioma stem cells that are CD133+ have been shown to be the cell of origin for infantile hemangiomas (35). These CD133+ cells differentiate into both the endothelial cells and the supporting mural cells in infantile hemangioma (35, 61, 74). Similarly, CD133+ cells have been identified in LMs which have progenitor characteristics such as pluripotency and recapitulated LMs in a xenograft model (26). CD133+ VMECs which display both progenitor and mature EC characteristics were sufficient to develop VM like channels lined by CD133+ and CD133- ECs similar to that observed in patient VM tissues (3). Thus, we have identified a novel cell type in VMs, a CD133+ EC that contributes to VM pathogenesis. Whether CD133+ VMECs give rise to CD133- VMECs or if these two cell types have distinct roles in VMs remains to be elucidated. Further studies of larger populations of VMECs will help improve understanding of VM genotype and phenotype correlations.

Presented at: the 62nd Plastic Surgery Research Council, Durham, North Carolina (May, 2017), 63rd Plastic Surgery Research Council, Birmingham, Alabama (May, 2018), the 22nd Workshop of the International Society for the Study of Vascular Anomalies Workshop, Amsterdam, Netherlands (May 2018).

## Data Availability

The original contributions presented in the study are included in the article/supplementary material, further inquiries can be directed to the corresponding author/s.
