# Supplementary material for: Identification of pathological CD133+ endothelial cells in venous malformations
Source: Front Cardiovasc Med. 2026 Mar 30;13:1760326. doi: 10.3389/fcvm.2026.1760326 (PMC13070831; doi:10.3389/fcvm.2026.1760326)
Supplement: Supplementary file 1 [file Table1.docx]

**Supplemental Figure 1. Expression of endothelial proteins at the surface of CD133- cells.** FACS analysis of CD31, VECADHERIN, VEGFR2, VEGFR3, CD146, CD34, and CD90 in HMVECs and CD133+ VM cells. Blue lines represent FACs with specific antibodies. Red lines represent IgG controls. HMVECs, human dermal microvascular endothelial cells; VM, venous malformation.

**Supplemental Figure 2. CD133+ and CD133- VM cells do not express the lymphocyte marker CD45.** CD45 FACS of HMVECs (control ECs), human chylothorax sample enriched in CD45+ lymphocytes, and CD133+ and CD133- VM cells. Blue or solid lines represent FACs with CD45 antibody. Red or dotted lines represent IgG control. ECs, endothelial cells; HMVEC, human dermal microvascular endothelial cells; VM, venous malformation.

**Supplemental Figure 3. pAKT^S473^ activation in CD133+ VMECs with *PIK3CA* and *TEK* variants A)** Mean pAKT^S473^ and **B)** nuclear pAKT^S473^ signal intensity normalized by number of HMVEC. Data presented for one experiment ± sem. ANOVA p<0.0001. Post-hoc Tukey test *p<0.0004, **p<0.0002, ***p<0.0001. HMVECs, human microvascular endothelial cells; pAKT, phosphorylated AKT.

**Supplemental Figure 4. Validation of human VECADHERIN antibody.** Human neonatal skin (n=3) and murine dermal tissues (n=3) were stained with a human specific VECADHERIN antibody or incubated without primary antibody (no 1^o^; negative control). White arrowheads highlight DAPI+ endothelial cells lining blood vessels. Scale bars 50 μm.

**Supplemental Figure 5. Control HMVEC xenografts. H**&E or VECADHERIN staining of HMVEC xenograft implants. Scale bars 100 μm. n = 4 xenografts were done.

**Supplemental Figure 6.** **Validation of CD133 antibody.** CD133 staining of VM tissue with and without primary antibody (no 1^o^; negative control). White arrowheads mark CD133+ cells. Yellow arrowhead mark ECs lining vascular channels. Scale bars 50 μm. VC, vascular channel.

**Supplemental Figure 7. Foreskin expresses PODOPLANIN and PROX1 on lymphatic vessels.** Foreskin was stained by immunofluorescence for PODOPLANIN (green) and PROX1 (red). Scale bar, 100 μm. V, vein, L, lymphatic.

**Supplemental Figure 8. Expression of PODOPLANIN proteins at the surface of VM39 CD133- cells.** PODOPLANIN FACS of VM 39 CD133- cells. Blue lines represent FACs with anti-PODOPLANIN antibody. Red lines represent IgG control. VM, venous malformation.

| **Supplemental Table 1: Summary of antibodies** | | | |
| --- | --- | --- | --- |
| **Antibody** | **Vendor (Cat No)** | **Dilution** | **Methodology** |
| CD31 | BD Biosciences (560984) | 1:43.5 | FACS |
|  | Dako (M0823) | 1:50 | Immunofluorescence |
| CD34 | BD Biosciences (550619) | 1:43.5 | FACS |
| CD90 | BD Biosciences (555595) | 1:43.5 | FACS |
| CD133 | Millipore (MAB4399-I) | 1:50 | Immunofluorescence |
| CD146 | Invitrogen (11-1469-42) | 1:43.5 | FACS |
| CD304 | Miltenyi Biotec  (130-090-533) | 1:11.2 | FACS |
| total AKT | Cell Signaling (9272) | 1:1000 | Western |
| pAKT | Cell Signaling (4060) | 1:1000 | Western/Immunofluorescence |
|  | Cell Signaling (2965) | 1:100 | Immunofluorescence |
| total ERK | Cell Signaling (4695) | 1:1000 | Western |
| pERK | Cell Signaling (4370) | 1:1000 | Western |
| b-ACTIN | ABclonal (AC038) | 1:1000 | Western |
| VECADHERIN | R&D Systems (FAB9381P) | 1:11.2 | FACS |
|  | R&D Systems (AF938) | 1:100 | Colorimetric IHC Immunofluorescence |
| VEGFR2 | R&D Systems (FAB357P) | 1:11.2 | FACS |
|  | R&D Systems (AF537) | 1:100 | Immunofluorescence |
| VEGFR3 | R&D Systems (FAB3492P) | 1:11.2 | FACS |
| PODOPLANIN | R&D Systems (AF3670) | 1:200 | Immunofluorescence |
| PROX1 | R&D Systems (AF2727) | 1:50 | Immunofluorescence |
| **Secondary Antibodies** | | | |
| Donkey Antimouse IgG  Alexa-Fluor 488 | Invitrogen A21202 | 1:1000 | Immunofluorescence |
| Donkey Antigoat IgG AlexaFluor 594 | Invitrogen A11058 | 1:1000 | Immunofluorescence |
| Donkey Antigoat IgG AlexaFluor 488 | Invitrogen A11055 | 1:1000 | Immunofluorescence |
| Donkey Antirabbit IgG Alexa-Fluor 594 | Invitrogen A212027 | 1:1000 | Immunofluorescence |
| Donkey Antisheep IgG Alexa-Fluor 488 | Invitrogen A11015 | 1:300 | Immunofluorescence |
| Rabbit Antigoat IgG Antibody,  Biotinylated | Vector Laboratories BA-5000 | 1:200 | Colorimetric IHC |

| **Supplemental Table 2. qRT-PCR Primer sequences** | | |
| --- | --- | --- |
| **Gene** | **Forward** | **Reverse** |
| β*-actin* | 5' CGA GGC CCA GAG CAA GAG AG 3' | 5' CTC GTA GAT GGG CAC AGT GTG 3' |
| *ANGPT1* | 5' GAC TGT GCA GAT GTA TAT CAA GCT G 3' | 5' TTT CAT TTC CTA TGT GGA ATC TGT C 3' |
| *ANGPT2* | 5' AGA AGA GAT CAA GGC CTA CTG TGA C 3' | 5' ATC ATT TCC TGG TTG GCT GAT G 3' |
| *CDH5/ VECADHERIN* | 5' TGT GAC AGC AGT GGA TGC AGA 3' | 5' CTG TAC TTG GTC ATC CGG TTC TG 3' |
| *KDR/VEGFR2* | 5' GGA CTG GCT TTG GCC CAA T 3' | 5' CTT GCT GTC CCA GGA AAT TCT G 3' |
| *FLT1/VEGFR1* | 5' GAA GGA GAG GAC CTG AAA CTG TC 3' | 5' ACT CTT TCA ATA AAC AGC GTG CTG 3' |
| *FLT4/VEGFR3* | 5' GAG ACC TGG CTG CTC GGA AC 3' | 5' TCA GCA TGA TGC GGC GTA TG 3' |
| *COUPTFII* | 5′-GCCATAGTCCTGTTCACCTC-3′ | 5′-CTGAGACTTTTCCTGCAAGC-3′ |
| *DLL4* | 5' CGG GTC ATC TGC AGT GAC AAC 3' | 5' AGT TGA GAT CTT GGT CAC AAA ACA G 3' |
| *EPHB4* | 5' CCA GAC TGT CCC ACC TCC CTC 3' | 5' GGC CAA GAT TTT CTT CTG GTG TC 3' |
| *EPHRINB2* | 5' AAA GAC CAA GCA GAC AGA TGC AC 3' | 5' CCA GCA GAA CTT GCA TCT TGT C 3' |
