# Supplementary figures and images for: Identification of pathological CD133+ endothelial cells in venous malformations
Source: Front Cardiovasc Med. 2026 Mar 30;13:1760326. doi: 10.3389/fcvm.2026.1760326 (PMC13070831; doi:10.3389/fcvm.2026.1760326)

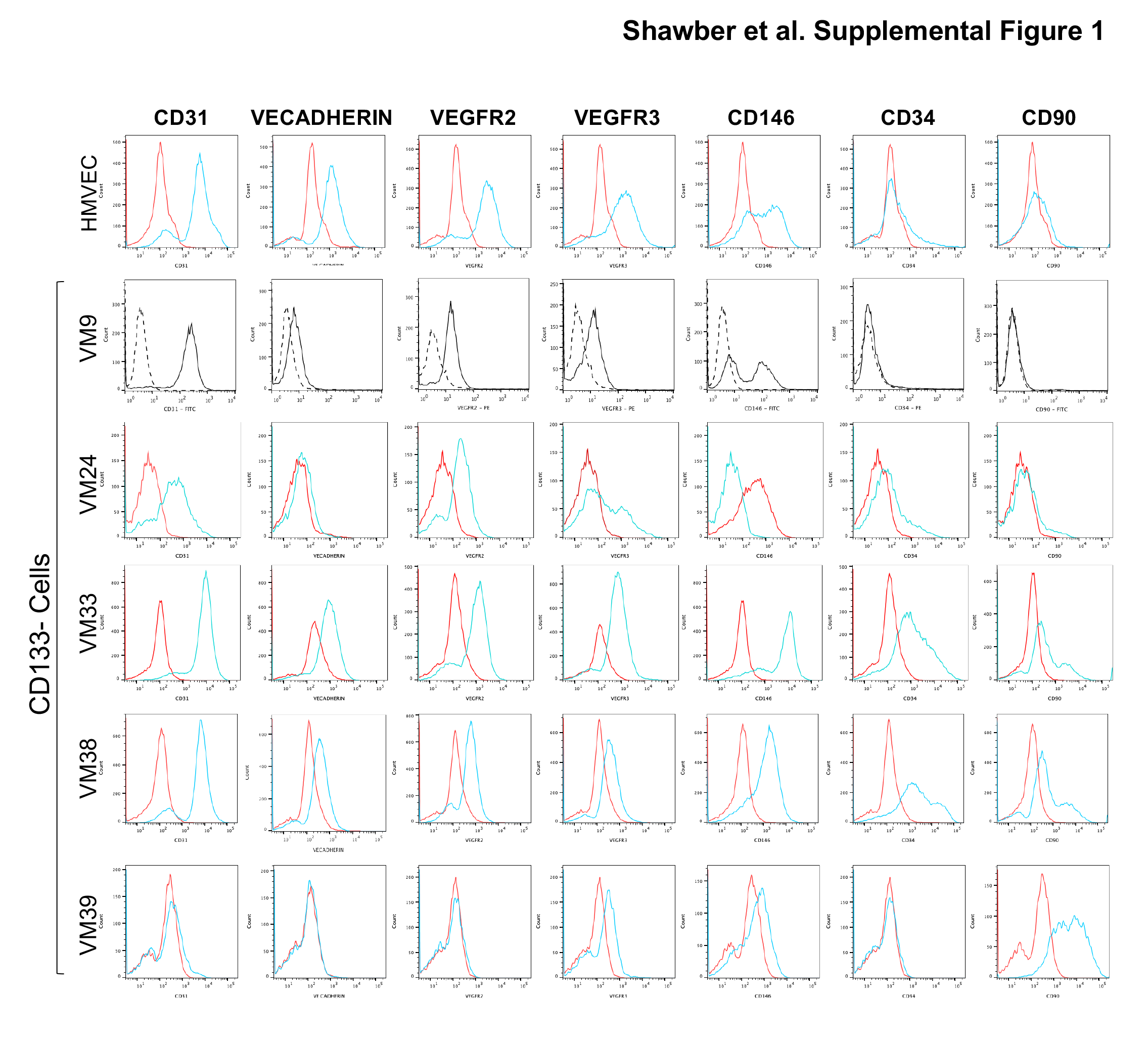

Supplement: Supplementary file 3 [file Image1.tif]

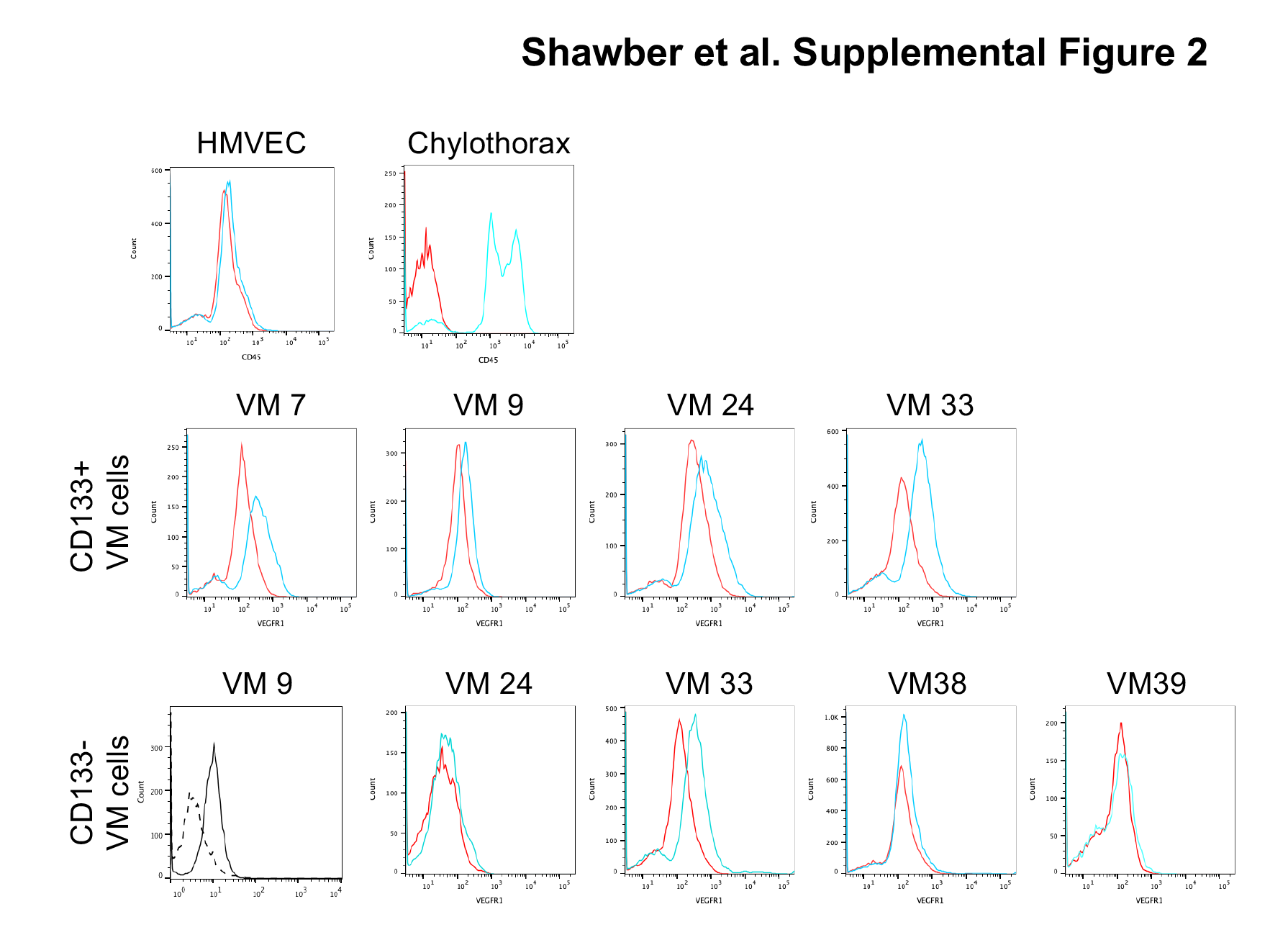

Supplement: Supplementary file 4 [file Image2.tif]

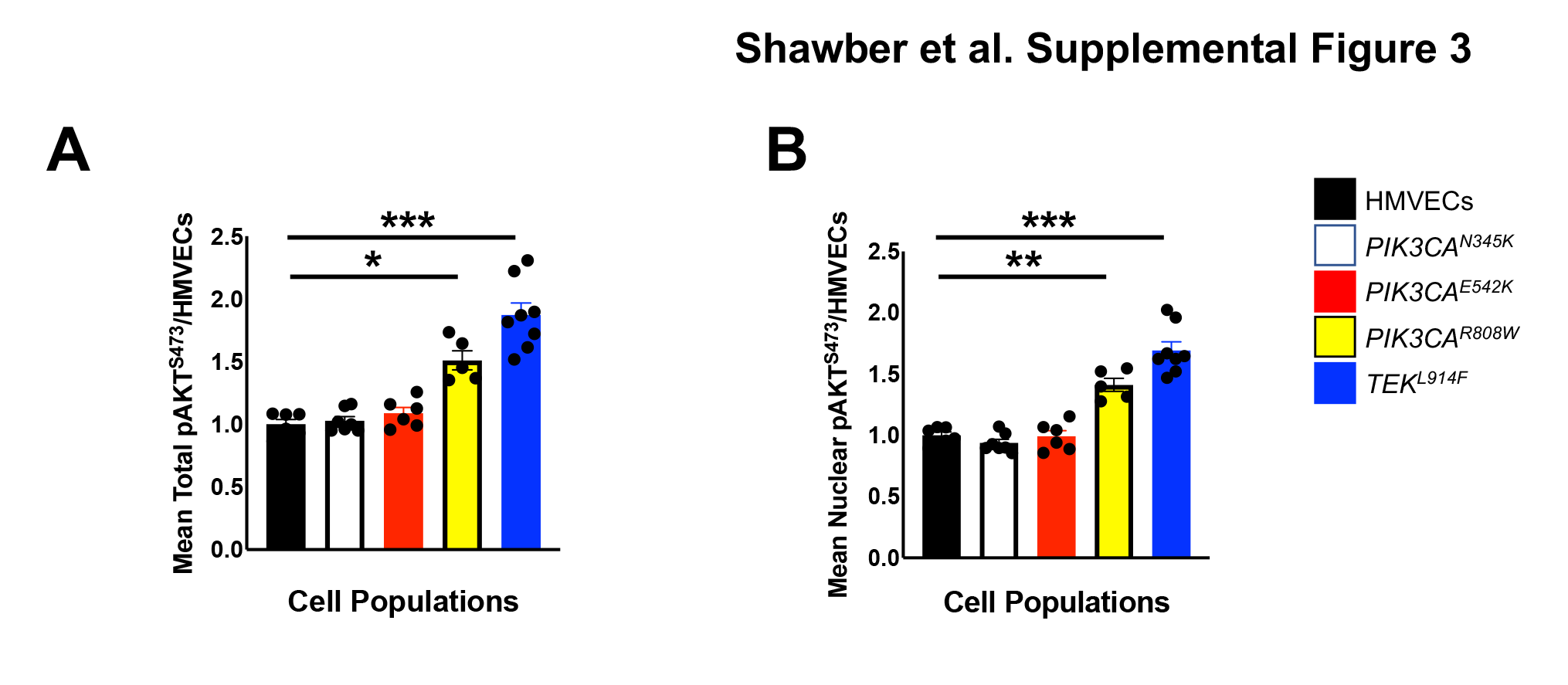

Supplement: Supplementary file 5 [file Image3.tif]

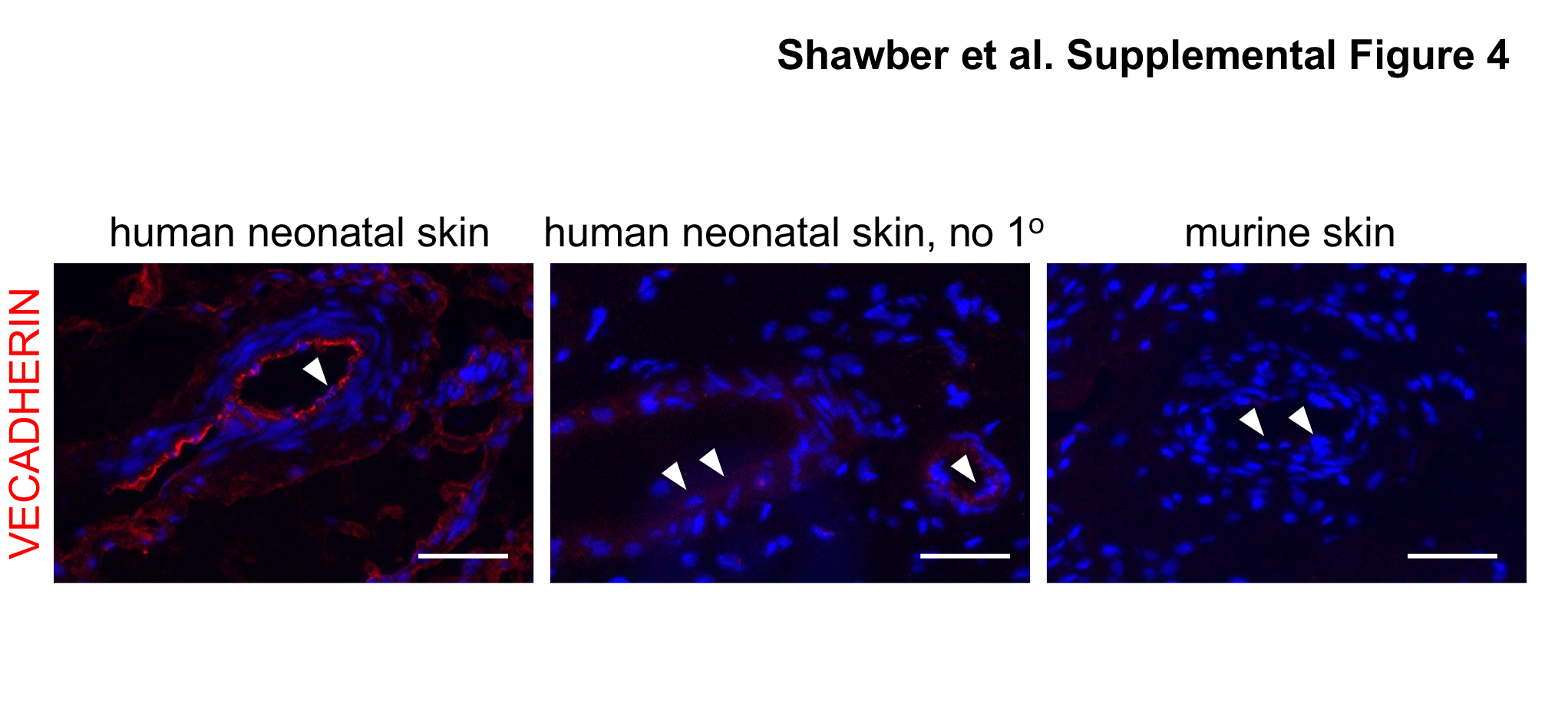

Supplement: Supplementary file 6 [file Image4.tif]

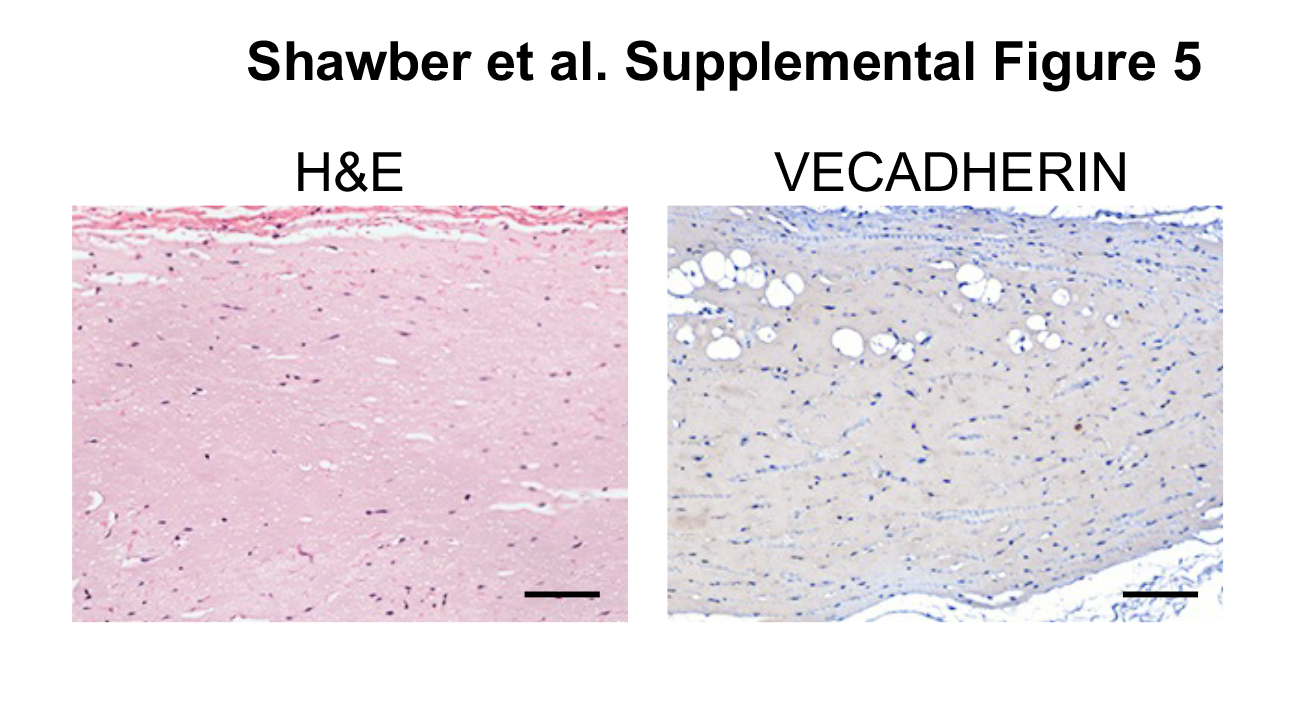

Supplement: Supplementary file 7 [file Image5.tif]

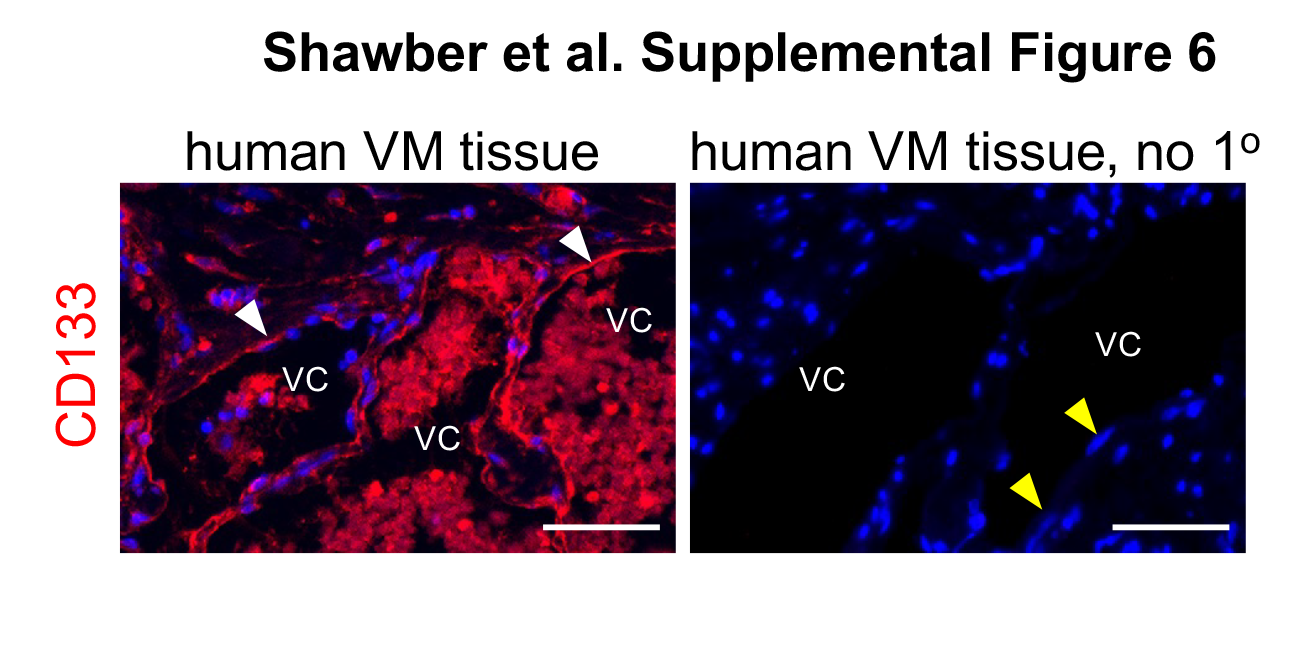

Supplement: Supplementary file 8 [file Image6.tif]

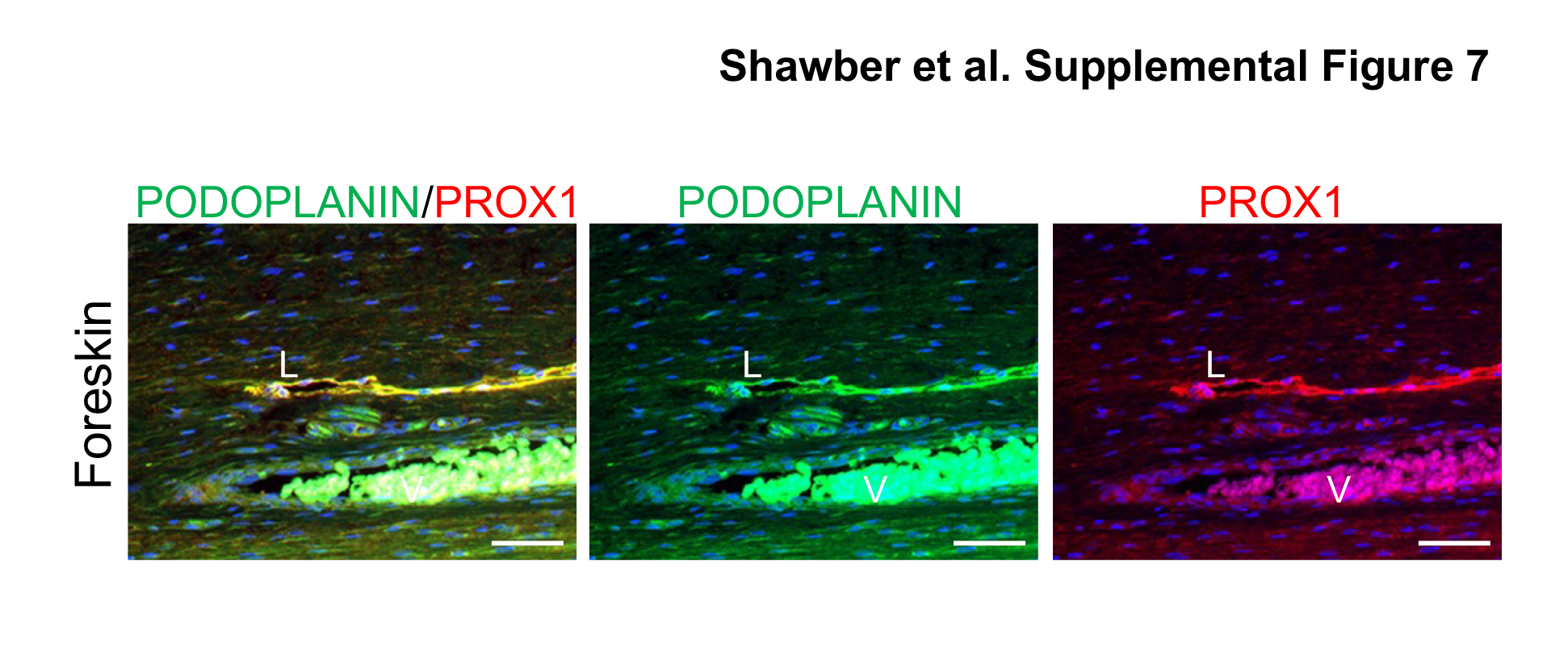

Supplement: Supplementary file 9 [file Image7.tif]

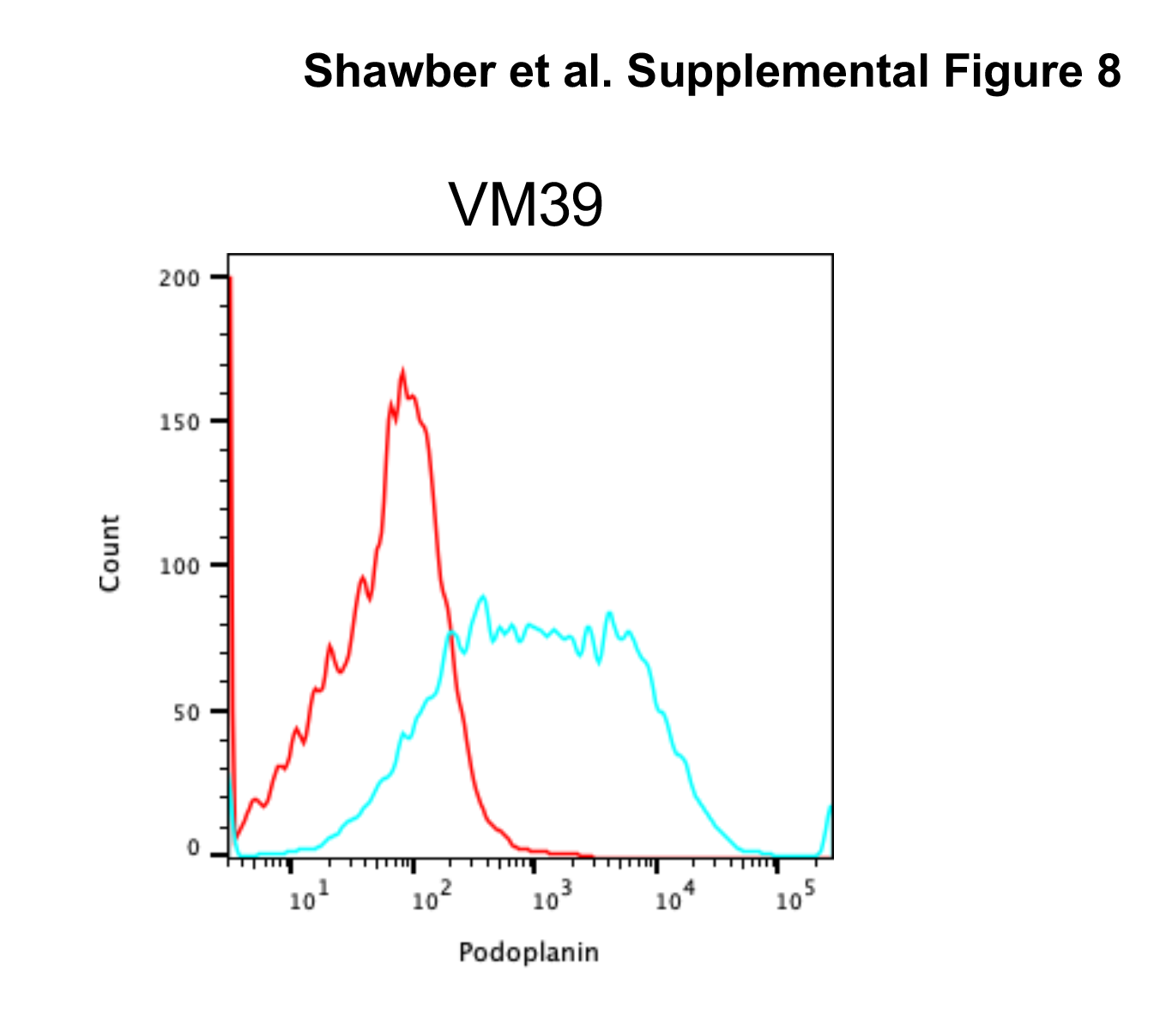

Supplement: Supplementary file 10 [file Image8.tif]
